# Mechanisms Underlying Lumbopelvic Pain During Pregnancy: A Proposed Model

**DOI:** 10.3389/fpain.2021.773988

**Published:** 2021-12-02

**Authors:** Catherine Daneau, Jacques Abboud, Andrée-Anne Marchand, Mariève Houle, Mégane Pasquier, Stephanie-May Ruchat, Martin Descarreaux

**Affiliations:** ^1^Department of Anatomy, Université du Québec à Trois-Rivières, Trois-Rivières, QC, Canada; ^2^Department of Human Kinetics, Université du Québec à Trois-Rivières, Trois-Rivières, QC, Canada; ^3^Department of Chiropractic, Université du Québec à Trois-Rivières, Trois-Rivières, QC, Canada

**Keywords:** hormonal changes, biomechanical changes, neuromuscular adaptations, motor control, pain modulation

## Abstract

Up to 86% of pregnant women will have lumbopelvic pain during the 3rd trimester of pregnancy and women with lumbopelvic pain experience lower health-related quality of life during pregnancy than women without lumbopelvic pain. Several risk factors for pregnancy-related lumbopelvic pain have been identified and include history of low back pain, previous trauma to the back or pelvis and previous pregnancy-related pelvic girdle pain. During pregnancy, women go through several hormonal and biomechanical changes as well as neuromuscular adaptations which could explain the development of lumbopelvic pain, but this remains unclear. The aim of this article is to review the potential pregnancy-related changes and adaptations (hormonal, biomechanical and neuromuscular) that may play a role in the development of lumbopelvic pain during pregnancy. This narrative review presents different mechanisms that may explain the development of lumbopelvic pain in pregnant women. A hypotheses-driven model on how these various physiological changes potentially interact in the development of lumbopelvic pain in pregnant women is also presented. Pregnancy-related hormonal changes, characterized by an increase in relaxin, estrogen and progesterone levels, are potentially linked to ligament hyperlaxity and joint instability, thus contributing to lumbopelvic pain. In addition, biomechanical changes induced by the growing fetus, can modify posture, load sharing and mechanical stress in the lumbar and pelvic structures. Finally, neuromuscular adaptations during pregnancy include an increase in the activation of lumbopelvic muscles and a decrease in endurance of the pelvic floor muscles. Whether or not a causal link between these changes and lumbopelvic pain exists remains to be determined. This model provides a better understanding of the mechanisms behind the development of lumbopelvic pain during pregnancy to guide future research. It should allow clinicians and researchers to consider the multifactorial nature of lumbopelvic pain while taking into account the various changes and adaptations during pregnancy.

## Introduction

Pregnancy-related low back pain (LBP) and/or pelvic girdle pain (PGP) are very common conditions, affecting up to 86% of pregnant women in the 3rd trimester of pregnancy (1). LBP is defined as “pain or discomfort located between the 12th rib and the gluteal fold,” and PGP as “pain experienced between the posterior iliac crest and the gluteal fold” (2). When both types of pain are present, the pain is frequently referred to as lumbopelvic pain (LBPP). Despite variation in definitions, there seems to be a consensus that the term LBPP is used when no distinction is made between LBP and PGP (3). The peak of LBPP intensity generally appears between 24 and 36 weeks of pregnancy (4). Women suffering from LBPP during pregnancy will still experience LBPP beyond 3 months [33% (5)] and 12 months [25% (3, 6)] after delivery.

Usually, women experience a decrease in health-related quality of life during pregnancy, but the decrease is greater for those with LBP (7). Indeed, pregnancy-related LBPP has important consequences on daily functioning and overall well-being (8–11). Importantly, LBPP has been found to contribute to high levels of sick leave in pregnant women (8).

## Hypothesis

Several potential risk factors for pregnancy-related LBP or PGP have been suggested; most of them being drawn from observational studies. Based on the exhaustive review of Verstraete et al. (12), history of LBP, previous trauma to the back or pelvis and previous pregnancy-related PGP seem to be strong risk factors for PGP during pregnancy. Two large population studies *n* = 74 973 (13) and *n* = 91 721 (14) also found that early menarche ( ≤ 13 years old), low maternal age (<35 years old), high body mass index (≥25 kg/m^2^), parity, low educational level ( ≤ 16 years of education), the presence of LBP before the first pregnancy, emotional distress, physically demanding work and the use of oral contraceptives were associated with increased odds for PGP during pregnancy. More specifically regarding oral contraception, Bjelland et al. (14) reported that the association between combined oral contraceptive pills and PGP during pregnancy was negative for primiparae (slightly protective effect of combined oral contraceptives) and positive for multiparae (marginally increased risk of PGP). Based on sub-analyses, the authors also concluded that long duration of exposure to a progestin intrauterine device or progestin-only oral contraceptives was associated with reduced odds of persistent pelvic girdle pain (Ptrend = 0.021 and Ptrend = 0.005, respectively). Conversely, long duration of exposure to progestin injections and/or a progestin implant was associated with modest increased odds of persistent pelvic girdle pain (Ptrend = 0.046). Finally, the authors reported that early timing of progestin-only contraceptive dispense following delivery (<3 months) was not significantly associated with persistent pelvic girdle pain (14).

Lack of exercise in mid-pregnancy has also been suggested to be associated with higher prevalence of LBP or PGP in late pregnancy (15).

Despite the high prevalence of pregnancy-related LBPP and its significant impact on women's quality of life, the physiological and biomechanical processes underlying the development of pregnancy-related LBPP remain unclear. This might explain why optimal management strategies that are based on underlying pathophysiological mechanisms are still lacking. In this hypothesis paper, we highlight several potential pregnancy-related changes (hormonal and biomechanical) and adaptations (neuromuscular) and propose a hypotheses-driven model describing how these various physiological and biomechanical changes potentially interact in the development of LBPP in pregnant women. A better understanding of the mechanisms behind the development of LBPP during pregnancy should guide future research and lead to more effective management.

## Lumbopelvic Pain Underlying Mechanisms

### Hormonal Changes

Pregnancy is characterized by significant hormonal changes, such as an increase in relaxin, estrogen and progesterone levels, and it has been hypothesized that these pregnancy-related hormones are potential contributors to LBPP development and intensity (16).

In pregnant women, relaxin levels increase by the end of the 1st trimester and remain relatively high until delivery (16). The role of relaxin is to relax spinal and pelvic ligaments and joints in order to facilitate childbirth. Relaxin has been shown to increase the laxity of the pubic ligaments in guinea pigs (17). A study showed that female guinea pigs that received hormonal treatment (relaxin or relaxin + estrogen) had weaker anterior cruciate ligaments (ACL), as reflected by a lower load capacity before failure for both hormonal treatments (compared to no treatment) and an increase in tibial displacement (compared to baseline), potentially suggesting knee instability (18). A review on the effect of relaxin on human and animal musculoskeletal structures, such as ligaments, suggested that relaxin could predispose the joints to non-traumatic injury via its effect on peripheral ligament laxity (19). As such, it has been suggested that relaxin may trigger spinal and pelvic instability, potentially inducing pain (19, 20). However, based on the findings of a systematic review, the association between relaxin levels and pregnancy-related LBPP is inconsistent and the quality level of the evidence is poor (21).

Compared to relaxin, studies investigating the effect of estrogen and progesterone on ligaments property and joint instability are conflicting. During pregnancy, estrogen levels raise steeply during the 3rd trimester (22). In addition to promoting fetal growth and well-being, estrogen has an effect on several properties of the musculoskeletal tissue such as bone, cartilage and ligaments, modulates the nervous system and potentially contributes to LBPP development and intensity (16). A study conducted on human ACL specimens showed that the higher the estrogen levels, the lower the fibroblasts proliferation and synthesis of procollagen, possibly increasing ligament laxity (23). This correlation was attenuated with increased progesterone levels (23). It has also been shown that high estrogen levels in the 3rd trimester of human pregnancy were correlated with an increase in anterior translation of the tibia, suggesting an increase in ACL laxity (24). However, a study reported that estrogen levels were not different between women reporting PGP and those not reporting such pain (4).

Finally, progesterone levels increase significantly throughout pregnancy, with a peak at ~15 weeks (25). This hormone contributes to the relaxation of all smooth muscles during pregnancy (22), with a possible effect on LBPP development and intensity (23). To the best of our knowledge, only one study investigated the possible association between progesterone and LBPP and reported that progesterone levels were significantly higher in the 1st trimester of pregnancy (6 to 12 weeks) among women reporting PGP compared to those reporting no pain (4).

Levels of prolactin and oxytocin also fluctuate during pregnancy and postpartum period. Prolactin, which increases in the 1st trimester, is 10 time higher at the end of pregnancy (26) and plays a major role in stimulating maternal milk production (27). Oxytocin level increases during pregnancy and reaches its peak at the very end (26). Oxytocin is a stimulator of uterine contractions during childbirth (27).

Based on current evidence, relaxin, estrogen, and progesterone may play a role in the development and intensity of pregnancy-related LBPP but further studies are needed to confirm this hypothesis. The role of prolactin and oxytocin remains to be investigated. The strength of correlation between hormonal status and LBPP development and intensity should be investigated through cohort studies.

### Biomechanical Changes

Profound biomechanical changes occur over the course of pregnancy (28). However, only a few studies investigated the association between these changes and LBPP. The most important pregnancy-related changes occur in the lower trunk and pelvic areas in response to the fetal load (29). Different kyphosis and lordosis configurations (30–37), as well as pelvic positions (30, 38, 39), have been described in response to the change in magnitude and distribution of loads acting on the spine during pregnancy. The overall increase in lumbar lordosis is generally heterogeneous and inconstant across studies. The post-partum period is also marked with variable modifications to spinal curvatures (33, 40–42) that appear to be independent of the ones observed during pregnancy. Despite this heterogeneity and inconsistency, most pregnant women experience a small absolute increase in lumbar lordosis and in LBP intensity, most commonly during the 2nd and 3rd trimesters, when the size of the fetus increases significantly and the hormonal milieu changes markedly (31, 33, 35, 43). Although the increase in lumbar lordosis angle and in LBP intensity follow parallel trajectories throughout pregnancy, the causality in this relationship remains unclear.

Several studies have examined the association between pre-pregnancy joint hypermobility and LBPP during pregnancy, with mixed results (44–47). Some studies reported an association (45–47), with an early study (47) showing a very strong relation between degree of symphyseal laxity and the risk for pelvic girdle pain during post-partum. However, a study with smaller sample size, found no association (44). Other authors suggested that pain could be explained by an asymmetric laxity of the sacro-iliac joints rather than by an increase in joint laxity *per se* (48). The joint laxity hypothesis is further emphasized by the fact that a possible cumulative effect exists as multiparity is a risk factor for PGP during pregnancy (13, 49).

Furthermore, sustained strain of pelvic structures and muscle weakness are believed to decrease mechanical force closure of the sacro-iliac joints, negatively influencing load transfer (50). Such proposed reduction in load transfer abilities are coherent with findings of altered gait pattern, including longer double limb support, shorter step length and less pelvic and hip movement, in women with pregnancy-related PGP (51–53). As previously mentioned, previous trauma to the back or pelvis area have been reported to be strong predictors of LBPP during pregnancy (12, 49) potentially leading to muscular imbalances and joint misalignments, which in turn could be aggravated by pregnancy-related anatomical changes (49). Finally, pregnancy-related morphological changes related to the bone structures of the pelvis, including increased pubic symphysis width and inter-ischial tuberosity distance, have been proposed as potential contributors to PGP (54–56).

### Neuromuscular Adaptations

Evidence suggesting motor control and neuromuscular adaptations during pregnancy and a possible link with LBPP is scarce, but some “patterns” of adaptations seem to be more consistent across studies (54). Several studies have identified an increased activation of lumbopelvic muscles during pregnancy. During a trunk flexion-extension task, pregnant women without LBPP show a significant increase in erector spinae muscle activation during the active flexion and the static full flexion phases of movement (57). Erector spinae and biceps femoris activity also seems to increase in upright posture in pregnant women compared to non-pregnant women (40). Compared to pregnant women without LBPP, those with LBPP have increased rectus femoris, abdominal obliques, psoas major, and adductor longus activities during active straight leg raising test (ASLR) (58) as well as increased intravaginal muscle activation during simulated pushing contractions (59). Stuge et al. (60) also reported a smaller levator hiatus (a proxy measure of pelvic floor muscle activation) at rest, during voluntary contractions and contraction associated to ASLR in women with PGP compared to those without PGP. Interestingly, an early study showed that pain intensity and erector spinae muscle activation were correlated in the 1st trimester of pregnancy, potentially suggesting a dose-response relationship (61).

Despite no clear evidence of neuromuscular manifestation of muscle fatigue, decrease endurance of the pelvic floor muscles, as well as an increased perineal tonus, have been reported in association with pregnancy-related PGP (50, 59, 62). Moreover, women with PGP and/or combined PGP and LBP were shown to have lower trunk muscle endurance (52).

Interestingly, two recently published studies (40, 57) reported that changes in trunk and lower limb muscle activation during upright standing and flexion-extension movements of the trunk are similar before and after pregnancy, suggesting progressive short-term neuromuscular adaptations during pregnancy that appear to rapidly fade following delivery. Reversibility in neuromuscular adaptations that occur during pregnancy is also supported by an earlier study indicating that reduced trunk ranges of motion during pregnancy return to pre-pregnancy values during the postpartum period (57).

### Pain Modulation

Central and peripheral sensitization have both been suggested to play a role in chronic pain conditions such as non-specific LBP and may increase the risk of a persistent pain. Physiological mechanisms leading to enhanced pain sensitivity have been thoroughly investigated and include sensitization of nociceptors and neuronal circuits (63), increased pain signaling through membrane excitability and synaptic efficacy, and inadequate descending pain inhibition mechanisms (64, 65). Whether or not these pain sensitization mechanisms play a significant role in the development and persistence of LBPP remains to be determined.

It has been consistently reported that women and men experience pain differently (66, 67). Women usually have a lower pain threshold and the cortical regions responsible for the affective component of pain show a higher brain activity (68). Prolonged pain can lead to central sensitization, which is also more present in women and mainly observed in women suffering from deep tissue pain (68). Differences in hormone levels between men and women represent the most important factor of these pain modulation disparities.

During pregnancy, changes in sex hormones, and the immune system improves to support the growing fetus (69). Sex hormones seem to play a role in pain modulation. Indeed, in non-pregnant women, it has been shown that an increase in estrogen levels correlates with a higher risk of LBP occurrence (70). During pregnancy, estrogen levels raise and have been hypothesized to contribute to LBPP development and intensity. Nevertheless, it has been suggested that hormone variations in pregnant women cannot be considered the sole contributor to anti-nociceptive or pro-nociceptive responses.

Interestingly, in female already living with chronic pain before becoming pregnant, an attenuation of pain symptoms has been observed during pregnancy. This adaptation refers to the pregnancy-induced analgesia phenomenon. This phenomenon has been reported for acute, as well as chronic, pain in pregnant animals and is dependent on various factors, such as variation in pregnancy-related hormone levels, the activation of cells of the immune system (T cells), and the release of δ-opioid (71, 72). However, the pregnancy-induced analgesia phenomenon is not always observed in humans. Studies have highlighted a progressive increase in pain threshold and tolerance in pregnant women compared to non-pregnant women (73, 74) while other studies failed to find such a pain adaptation (74–78). It has also been reported that widespread deep-tissue pressure hypersensitivity is higher and increased in pregnant women who experienced either low or high levels of LBPP compared to pain-free non-pregnant women (79). The discrepancy between animal and human studies on pain modulation could be explained by different factors, such as differences in pain threshold, location of the painful stimulus, age, medication and psychological aspects [fear-avoidance and catastrophizing of pain (80, 81)] which are more prevalent in humans (82).

At 4 to 8 weeks after delivery, an increase in hormone levels (i.e., estrogen and progesterone) has been observed in comparison to the last weeks of pregnancy (83). However, this hormone change does not seem to affect the pain modulation as similar heat pain threshold and tolerance in the upper limb were observed in postpartum women compared to pregnant (75) [and non-pregnant women (84)]. Yet, another study showed that postpartum women were more sensitive to pain at the rectus abdominis muscle than non-pregnant women (85). Conversely, lower capsaicin-evoked upper limb pain has been shown in postpartum women compared to non-pregnant women (84). Such postpartum analgesic phenomenon may be explained by oxytocin released into the systemic circulation and central nervous system, as evidenced by increased concentrations in cerebrospinal fluid during labor (86). Oxytocin may therefore play a role in pain modulation through reduced sensitization or enhanced inhibition mechanisms (84).

## Discussion

The past decades have seen a significant growth in mechanistic research aimed at investigating the physiological processes involved in the development and chronification of LBP. Although uncertainties persist, our understanding of biomechanical, motor control and neuromuscular adaptations to LBP has greatly improved and we now know that changes in motor behavior associated to LBP involve complex (87, 88) and most likely non-stereotypical “adaptive strategies” (89). Overall, LBP triggers adaptations at multiple levels of the motor system (87) which involves a redistribution of muscular activity within and between muscles as well as numerous changes in mechanical behaviors. According to “the new pain adaptation theory” proposed by Hodges and Tucker, and largely inspired by LBP research, the changes observed in the motor system occur in response to pain and are tailored to protect from further pain or re-injury, but also entail possible long-term adverse consequences. Whether pregnancy-related LBPP and non-specific LBP share similar underlying physiological mechanisms is still up for debate but the fact that pregnant women go through several physical and biomechanical adaptations in a relatively short period of time is unmistakable.

**Figure 1:**
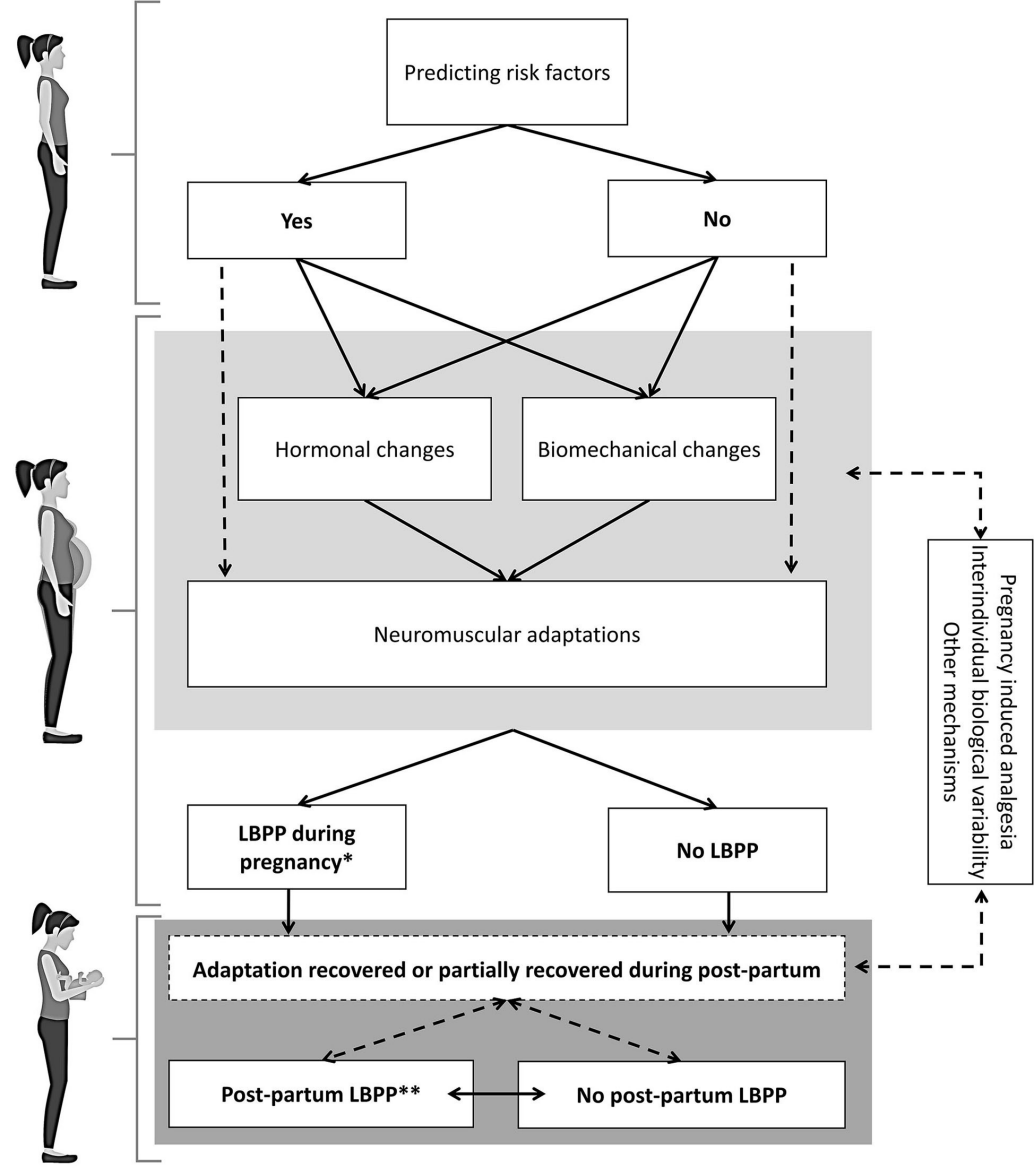
Lumbopelvic pain (LBPP) underlying mechanisms. This model presents the potential pregnancy-related hormonal and biomechanical changes as well as neuromuscular adaptations that may play a role in the development and intensity of LBPP in pregnant women. Dotted arrows indicate potential modulating factors or hypothetical relationship between two boxes. *The peak of LBPP intensity generally appears between 24 and 36 weeks of pregnancy (4). **Women suffering from LBPP during pregnancy will still experience LBPP beyond 3 months [33% (5)] and 12 months [25% (3, 6)] after delivery.

Similarly to LBP, LBPP is a complex and multifaceted condition for which underlying mechanisms remain elusive. The development and evolution of LBPP during and following pregnancy are probably dictated by complex interactions between risks factors, physiological processes and other contextual factors. The following theoretical model will hopefully provide some insights into these complex interactions and guide the design and interpretation of both mechanistic and clinical research. The model, shown in Figure 1, postulates that every pregnant woman can have a varying number of LBPP risk factors. During pregnancy, women will undergo several changes and adaptations that can be potentially mediated by other individual characteristics and biological mechanisms such as changes in pain modulation. Interactions between these various changes and adaptations remain mostly to be investigated. Our research team is currently conducting a prospective laboratory study which aims to characterize the evolution of neuromechanical (lumbar muscle activity and kinematic), physiological (pregnancy-related hormones) and clinical (pain intensity and disability) changes at all pregnancy trimesters.

The fact that the several physiological changes occurring during pregnancy, including motor behaviors and neuromuscular control, are not systematically associated to LBPP (or the threat of LBPP) suggests that motor adaptations during pregnancy may precede LBPP. However, one must not forget that previous history of LBP is one of the most powerful LBPP predictor. Following delivery, women fully or partially return to pre pregnancy hormonal, biomechanical and neuromuscular state and may or may not experience persistent or recurring LBPP in the post-partum period. Whether or not recovering from pregnancy-related changes and adaptations can explain persisting LBPP remains to be investigated.

## Conclusion

In conclusion, we proposed a model for underlying causes of LBPP that should allow clinicians and researchers to consider the multifactorial nature of LBPP and the potentially competing mechanisms (biomechanical, hormonal, and neuromuscular processes) as well as their interactions. It also considers and weighs in current evidence to guide future research. Future research should include observational studies conducted to determine the potential role of individual and combined physical and physiological adaptations in the development of LBPP in pregnant women.

## Data Availability Statement

The original contributions presented in the study are included in the article/supplementary material, further inquiries can be directed to the corresponding author/s.

## Author Contributions

CD, S-MR, and MD contributed to conceptualization. CD, JA, S-MR, and MD designed the model. S-MR and MD supervised the project. A-AM, S-MR, and MD revised the manuscript critically. All authors wrote the manuscript and approved the final manuscript.

## Funding

Funding for this study was provided by the Chaire de Recherche Internationale en Santé Neuromusculosquelettique and its partner, the Centre Intégré Universitaire de Santé et de Services Sociaux de la Mauricie-et-du-Centre-du-Québec.

## Conflict of Interest

The authors declare that the research was conducted in the absence of any commercial or financial relationships that could be construed as a potential conflict of interest.

## Publisher's Note

All claims expressed in this article are solely those of the authors and do not necessarily represent those of their affiliated organizations, or those of the publisher, the editors and the reviewers. Any product that may be evaluated in this article, or claim that may be made by its manufacturer, is not guaranteed or endorsed by the publisher.
